# Association between Grape Yeast Communities and the Vineyard Ecosystems

**DOI:** 10.1371/journal.pone.0169883

**Published:** 2017-01-13

**Authors:** João Drumonde-Neves, Ricardo Franco-Duarte, Teresa Lima, Dorit Schuller, Célia Pais

**Affiliations:** 1 CITAA—Research Center for Agricultural Technology of Azores, University of Azores, Angra do Heroísmo, Portugal; 2 Centre of Molecular and Environmental Biology (CBMA), Department of Biology, University of Minho, Braga, Portugal; University of Torino, ITALY

## Abstract

The grape yeast biota from several wine-producing areas, with distinct soil types and grapevine training systems, was assessed on five islands of Azores Archipelago, and differences in yeast communities composition associated with the geographic origin of the grapes were explored. Fifty-seven grape samples belonging to the *Vitis vinifera* grapevine cultivars Verdelho dos Açores (*Verdelho*), Arinto da Terceira (*Arinto*) and Terrantez do Pico (*Terrantez*) were collected in two consecutive years and 40 spontaneous fermentations were achieved. A total of 1710 yeast isolates were obtained from freshly crushed grapes and 1200 from final stage of fermentations. Twenty-eight species were identified, *Hanseniaspura uvarum*, *Pichia terricola* and *Metschnikowia pulcherrima* being the three most representative species isolated. *Candida carpophila* was encountered for the first time as an inhabitant of grape or wine-associated environments. In both sampling years, a higher proportion of *H*. *uvarum* in fresh grapes from *Verdelho* cultivar was observed, in comparison with *Arinto* cultivar. Qualitatively significant differences were found among yeast communities from several locations on five islands of the Archipelago, particularly in locations with distinctive agro-ecological compositions. Our results are in agreement with the statement that grape-associated microbial biogeography is non-randomly associated with interactions of climate, soil, cultivar, and vine training systems in vineyard ecosystems. Our observations strongly support a possible linkage between grape yeast and wine typicality, reinforcing the statement that different viticultural terroirs harbor distinctive yeast biota, in particular in vineyards with very distinctive environmental conditions.

## Introduction

Traditionally, wines are produced by spontaneous fermentation carried out by the yeast biota naturally present in musts, having its origin on the grapes and/or winery equipment, and the process involves the sequential development of different yeast species. Strains of *Saccharomyces cerevisiae*, especially adapted, play the major role but the initial fermentation stages are usually carried out by non-*Saccharomyces* species [1]. The species *Hanseniaspora uvarum* (anamorph *Kloeckera apiculata*) is widely reported as predominant in initial stages of spontaneous fermentations, together with *Candida* spp. and *Pichia* spp. [2–9]. It is well known that the yeasts species/strains present during must fermentation affect wine’s flavor and aroma [10–14], because they act differently on musts, yielding different metabolites in different amounts [15–19]. Yeast biodiversity in vineyards are mainly affected by the grapevine cultivar [20–22], viticultural and oenological practices [23–29], macro and microclimatic conditions [1,30–32] and the geographic location of the vineyard [20,33–35]. It has been generally thought that different regions and grapevine cultivars, under different farm management practices–different viticultural *terroirs*–may harbor distinctive yeast communities and populations. Preserving the *terroir* characteristic of each wine has been one of the main concerns of the winemaking industry [21]. This led to an increased focus on the selection of autochthonous yeast, that might be better adapted to the fermentation of a particular grape must and might contribute to the typical oenological characteristics of a particular region [36–41]. Despite the suggestion that grape heath status is the main factor affecting the microbial ecology of grapes. [42] the idea that microbiological resources might be influenced by *terroir* aspects has long been suggested by several authors [21,22,43–45], and was recently demonstrated by using advanced short-amplicon sequencing approach by Bokulich et al. [32]. Moreover, the evidence for regional dispersion of vineyard-associated yeasts was previously described for cultivable yeast communities in New Zealand vineyards [46].

On the islands of the Azores Archipelago unique viticultural environments occur in several locations, resulting from the interaction between very particular macro and microclimatic conditions, autochthonous grapevine cultivars and local viticultural practices. Two important wine-producing regions in Azores Archipelago are Lajidos (PLJ) in Pico Island (classified by UNESCO as world heritage (http://whc.unesco.org/en/list/1117)) and Biscoitos (BCT), in Terceira Island, both corresponding to viticultural areas that are very distinct from the remaining locations in the archipelago.

Besides the vineyards installed in arable lands, grapevines were traditionally planted in poor soils (shallow or stony). Plants are placed in the irregularly distributed cracks of almost unmodified solidified lava flows (*lajido*) or in soils covered by thick layers of stones (*biscoito*). These particular types of soil ensure unique microclimates at the grape berry level, characterized by lower humidity and higher temperatures at maturation time. The grapevine training systems are mainly dependent on the soil type of the vineyard. Both in *biscoito* and in *lajido*, grapevines are not trellised, and the training system is unique for each plant depending on the area they have available. In the vineyards planted in arable soil, grapevines can be trellised or not.

The existence of different viticultural conditions in island environment, with well-defined borders, represents a suitable model to address the question of yeast biota / *terroir* association. Therefore, the aim of this study was to characterize the yeast biota from the Azorean traditional grape cultivars growing in several wine-producing areas from the Archipelago, and search for differences in grape yeast communities associated with those agro-ecological zones.

## Material and Methods

### Sampling and yeast isolation

**Fig 1:**
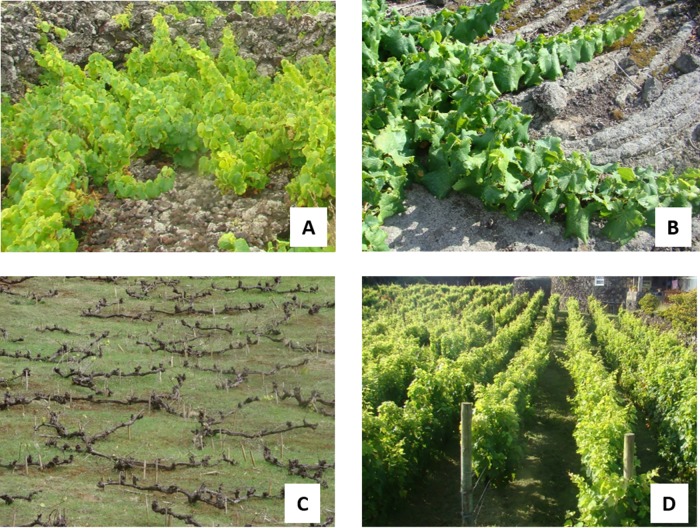
Combinations of soil type and grapevine training system in the sampled vineyards. A: not- trellised grapevine (NT) in soil covered with stones (SS); B: NT in solidified lava flows (SL); C: NT in arable soil (AS); D: trellised grapevines (TG) in AS.

The sampling areas were selected based on the combination of three agro-ecological criteria: *i)* type of soil (arable soil—*arable*; soil covered with stones—*stony*; solidified lava flows—*lava*); *ii)* grapevine cultivars (*Verdelho*: Verdelho dos Açores; *Arinto*: Arinto da Terceira; *Terrantez*: Terrantez do Pico); and *iii)* the grapevine training system (trellised grapevines, non-trellised grapevines; lying grapevines) (Fig 1).

**Fig 2:**
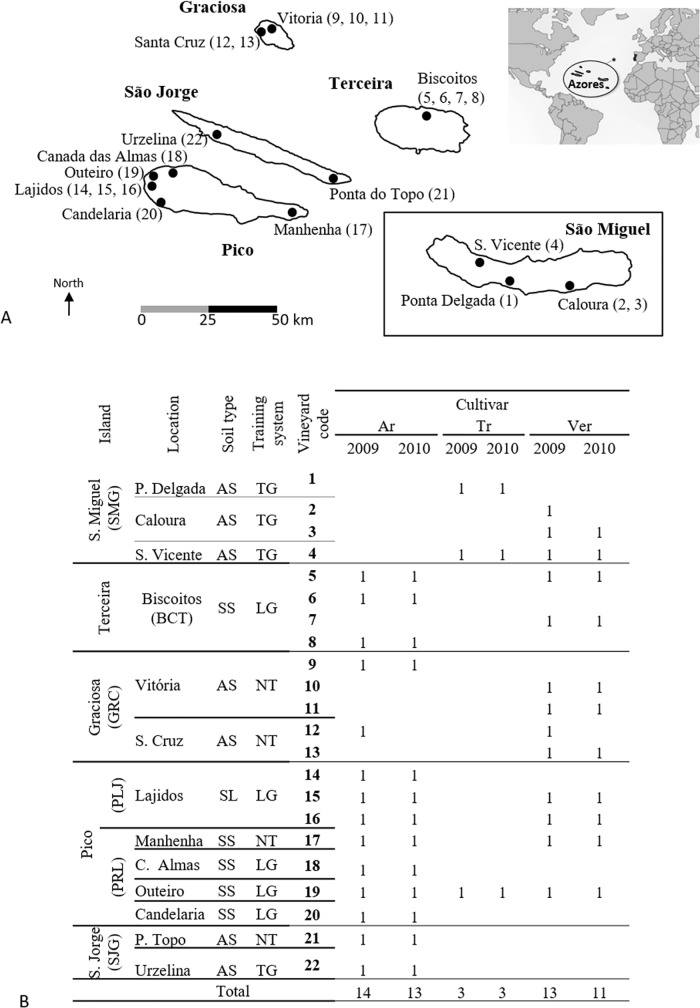
Sampling sites (A) and summary of the samples collected (B) in 2009 and 2010 in 22 vineyards from 13 wine-producing areas on five islands of the Azores Archipelago, from the grapevine cultivars Verdelho dos Açores (Ver), Arinto da Terceira (Ar) e Terrantez do Pico (Tr) planted in arable soil (AS), soil covered with stone (SS) or solidified lava flows (LF), and trained as trellised grapevines (TG), non-trellised grapevines (NT) or lying grapevines (LG).

Grape samples belonging to white *Vitis vinifera* cultivars *Verdelho*, *Arinto* and *Terrantez* were collected from 22 vineyards on 13 locations on five islands of the Azores Archipelago during the 2009 and 2010 harvests (30 and 27 grape samples, respectively) (Fig 2), always with the permission of the land owners. Locations were chosen within the existing vineyards, being the number of locations per island independent of its area. Each sample consisted of approximately 2–3 kg of rot-free grape bunches that were collected aseptically into sterile plastic bags and immediately transported to the laboratory under refrigerated conditions. For each location grape bunches were harvested in four different sampling points, separated by an average distance of *ca*. 10 m, in order to obtain a high diversity inside each harvest location. The berries were manually crushed inside the sterile bags, and from each sample 500 mL of must were obtained and fermented, using 500 mL Erlenmeyer flasks with a rubber stopper that was perforated with a syringe needle to allow CO_2_ release. Fermentations were performed at room temperature and progress was followed by daily weight loss determinations due to CO_2_ production. Immediately after the preparation of the must, diluted aliquots (10^−1^ to 10^−5^) were spread on plates containing YPD medium (yeast extract, 1% w/v; peptone, 1% w/v; glucose 2% w/v and agar 2%, w/v) supplemented with biphenyl (40 mg L^-1^). After incubation (2 days, 30°C), 30 colonies were randomly collected from plates containing between 30 and 300 colonies, which corresponded to a dilution of 10^−2^. When the weight loss of the must was about 65–70 g, corresponding to a stage close to the end of fermentation, must aliquots were again withdrawn, diluted and spread onto agar plates containing YPD medium (without biphenyl supplementation). Thirty colonies were randomly collected from plates containing between 30 and 300 colonies. Yeast isolates were stored in glycerol (30%, v/v) at -80°C.

### Molecular identification of the yeast isolates

DNA extraction was performed according to Drumonde-Neves *et al*. [47]. Molecular identification of isolates from freshly crushed grapes was performed by restriction fragment length polymorphism analysis (RFLP) and DNA sequencing. The 5.8-S ITS region was amplified using the primers ITS1 (5'-TCCGTAGGTGAACCTGCGG-3') and ITS4 (5'-TCCTCCGCTTATTGATATGC-3') [48]. PCR reaction was performed as follows: initial denaturation at 95°C for 6 min; 35 cycles of denaturing at 95°C for 20 s, annealing at 53°C for 20 s, extension at 72°C for 1 min; final extension at 72°C for 5 min. PCR amplification was carried out in a final volume of 10 *μ*L of a reaction mix containing 20–50 ng of yeast DNA, 0.5 U Taq polymerase (MBI Fermentas), 1x Taq buffer (10 mM TrisHCl, 50 mM KCl, 0.08% Nonidet P-40), 0.4 *p*mol of each primer, 0.2 mM of each deoxynucleotides and 1.5 mM MgCl_2_. After dilution (1:4), 10 *μ*L of the PCR products were digested with the restriction endonuclease *Hinf*I (Fermentas) according to the supplier’s instructions. PCR products and their restriction fragments were mixed and separated in a 2% (w/v) agarose gel containing GelRed^TM^, (1x TAE Buffer, 100 V, 75 min). Identical electrophoretic profiles of each sample were considered as conspecific and grouped, and one representative isolate per group was selected for sequencing of the 5.8-S ITS region. The amplicons were obtained as described above and sequenced by the Sanger method [49, 50]. Sequence reactions were performed by use of the forward primer ITS1, and a BigDye Terminator Cycle Sequence Ready Reaction Kit version 3.1 (Applied Biosystems, Foster City, CA). After the sequence reaction, excess dye terminators were removed by gel filtration. Sequences were analyzed with an automated DNA sequencer 3730XL (Applied Biosystems). Species identity was determined using the BLASTN program [51] and GenBank reference sequences, considering an identity threshold of at least 98%.

Regarding final stages of fermentation, all isolates were analyzed by interdelta sequence typing [52,53]. Isolates that showed no interdelta pattern were considered to belong to non-*Saccharomyces* species, and were identified using the method described above.

### Statistical analysis

Differences in community composition between islands/regions were tested by analysis of similarity (ANOSIM) on squared root transformed species incidences, using the informatics program PAST [54]. An analysis of similarity (ANOSIM) test makes no assumption about the normality of data and this multivariate test is classically used in community ecology. The test reports the probability of observing differences in community composition between islands/locations by chance using permutations of a rank Bray–Curtis similarity matrix to create null distributions [55]. The observed rank abundance difference (*R*) between islands/locations is reported, and this value ranges from -1 to +1. *R*-values above or below zero indicate that communities differ or not, respectively, between islands/locations. Null distributions were generated, that recalculated *R* under a framework that assumed no difference between regions, by randomizing the labels associated with samples across the entire data set one million independent times.

## Results

### Yeast species occurring on freshly crushed grapes

A total of 22 vineyards were sampled, spanning the geographic range of the wine-producing regions of the Azores Archipelago, as listed in Fig 2. Fifty-seven grape samples of the white grapevine cultivars Verdelho dos Açores, Arinto da Terceira and Terrantez do Pico were collected in 2009 and 2010, and 1710 yeast isolates were obtained from the freshly crushed grapes and analyzed by ITS-RFLP and by sequencing the 5.8-S ITS region. As summarized in Table 1 a total of 26 species were found in both years (14 in 2009 and 12 in 2010), corresponding to 19 different species since seven of them were found in both sampling years.

**Table 1:** Incidence of each yeast species (%) isolated from 57 grape samples collected on 5 islands of the Azores Archipelago (BCT: Biscoitos; GRC: Graciosa Island; PLJ: Lajidos; PRL: remaining locations in Pico; SJG: S. Jorge Island; SMG: S. Miguel Island) on freshly crushed grapes.

	Arinto da Terceira										Verdelho dos Açores										Terrantez do Pico			
	2009					2010					2009					2010					2009		2010	
	BCT	GRC	PLJ	PRL	SJG	BCT	GRC	PLJ	PRL	SJG	SMG	BCT	GRC	PLJ	PRL	SMG	BCT	GRC	PLJ	PRL	SMG	PRL	SMG	PRL
N° of grape samples	3	2	3	4	2	3	1	3	4	2	3	2	4	2	2	2	2	3	2	2	2	1	2	1
N° of yeast isolates	90	60	90	120	60	90	30	90	120	60	90	60	120	60	60	60	60	90	60	60	60	30	60	30
N° of species	3	5	4	5	5	5	2	7	3	7	2	4	4	5	3	3	4	3	3	1	4	3	4	3
*Barnettozyma californica*													0.80											
*Candida carpophila*														1.7										
*Candida inconspicua*																						30.0		
*Candida quercitrusa*										3.3														
*Candida railenensis*																							8.3	
*Hanseniaspora opuntiae*													49.2											
*Hanseniaspora uvarum*	1.1	48.3	1.1	21.7	70.0	53.3	96.7	64.4	98.3	70.0	73.3	35.0	44.2	11.7	83.3	96.7	95.0	91.1	78.3	100	85.0		88.3	90.0
*Hanseniaspora vineae*				0.8										1.7							1.7			
*Issatchenkia hanoiensis *										5.0							1.7							
*Metschnikowia pulcherrima*		3.3	95.6	37.5	3.3								5.8	83.3	15.0									
*Pichia cecembensis*		1.7	1.1					23.3		10.0					1.7									
*Pichia fermentans*								2.2																
*Pichia kudriavzevii*								1.1																
*Pichia membranifaciens*							3.3			1.7											10.0			
*Pichia terricola*	80.0	8.3	2.2	15.0	23.3	2.2		2.2	0.8	5.0		33.3		1.7		1.7		7.8	15.0			23.3		6.7
*Saccharomycopsis vini*					1.7	8.9		5.6	0.8	5.0						1.7	1.7	1.1	6.7				1.7	3.3
*Saccharomycopsis crataegensis*					1.7	5.6		1.1				1.7					1.7							
*Starmerella bacillaris*	18.9	38.3		25.0		30.0					26.7	30.0									3.3		1.7	
*Zygoascus meyerae*																						46.7		

Globally, the most representative species was *Hanseniaspora uvarum*, found in the three grape cultivars and in all islands, corresponding to 41% and 87% of the total isolates obtained in 2009 and 2010, respectively. In the second sampling year the incidence of this species was considerably higher in comparison to the previous year, ranging between 53.3% (grape samples from *Arinto* cultivar collected in Biscoitos) and 100% (grape samples from *Verdelho* collected in the remaining locations in Pico). *Metschnikowia pulcherrima* was the second most representative yeast species. However it occurred only in 2009 and with a considerably higher incidence on Lajidos (95.6% and 83.3%, *Arinto* and *Verdelho* grape cultivars, respectively) compared to Graciosa (3.3% and 5.8%, *Arinto* and *Verdelho* grape cultivars, respectively) and S. Jorge (3.3%, *Arinto* grape cultivar). *Pichia terricola* was the third most representative species, corresponding to 15% and 3% of the isolates obtained in 2009 and 2010, respectively. In the first sampling year this species was isolated from *Arinto* grapes in all islands, ranging between 2.2% and 80.0% of the isolates from Lajidos and Biscoitos, respectively. From the grape cultivar *Verdelho*, *P*. *terricola* was isolated in 2009 only in Biscoitos and Lajidos (33.3% and 1.7% of the isolates, respectively). In grape samples from the cultivar *Terrantez*, *P*. *terricola* was found only in “remaining locations in Pico” both in 2009 (23.3%) and 2010 (6.7%). In 2010 this species was also found in grape samples from *Arinto* collected in Biscoitos (2.2%), Lajidos (2.2%), “remaining locations in Pico” (0.8%) and S. Jorge (5.0%) and in samples from *Verdelho* collected in S. Miguel (1.7%), Graciosa (7.8%) and Lajidos (15%). Similar patterns of distribution among the different islands and sampling years were also observed for the yeast species with a lower global representation such as *Starmerella bacillaris* (former *Candida zemplinina*) or *Saccharomycopsis vini*. To our knowledge, the species *Candida carpophila* was not previously found in grape or wine-associated environments, unlike the remaining species listed in Table 1.

**Fig 3:**
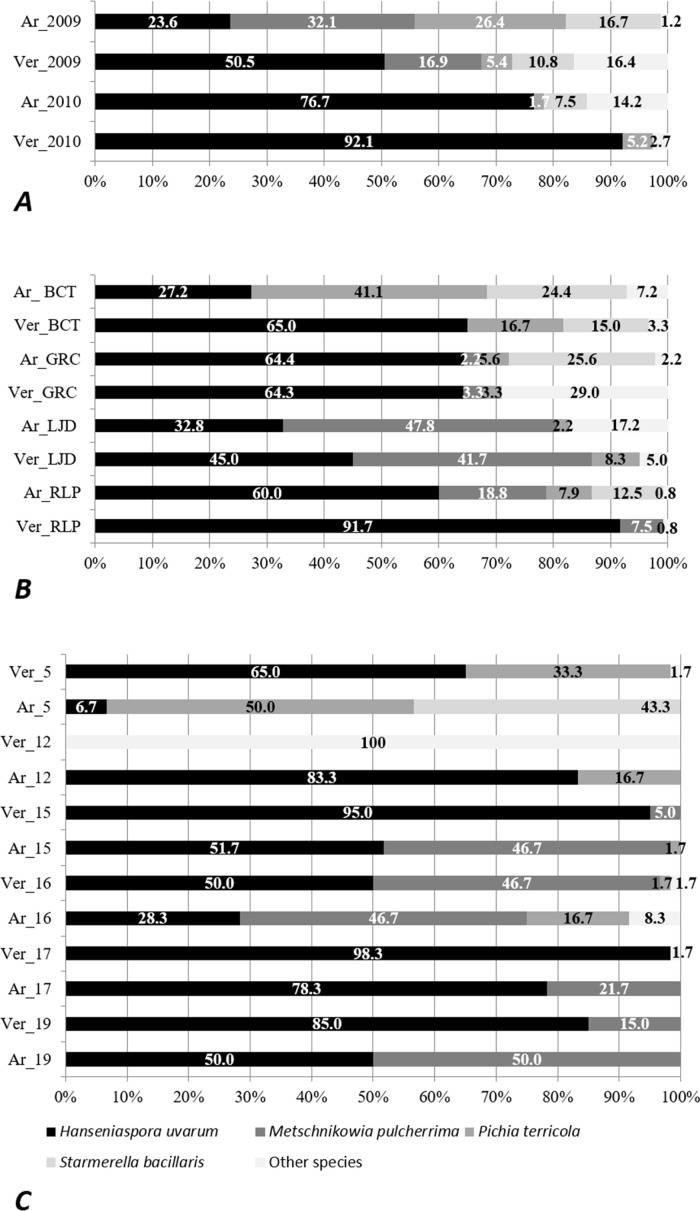
Yeast communities composition (in percentage) from freshly crushed grapes of the grapevine cultivars Verdelho dos Açores (Ver) and Arinto da Terceira (Ar) collected in 2009 and 2010 on A) all sampled locations, B) in Lajidos (PLJ), the remaining locations of Pico (PRL), in Biscoitos (BCT) and in Graciosa (GRC) and C) in six vineyard (numbers 5, 12, 15, 16, 17 and 19; see Table 1).

When analyzing the global yeast biota isolated from the grape samples of each of the cultivars (Fig 3A), we observed that the percentage of *H*. *uvarum* isolated from the *Verdelho* samples was higher compared to the samples from *Arinto* cultivar (26,9% and 15,4%, in 2009 and 2010, respectively). This same tendency was observed when the comparison of the yeast biota isolated from each of the two grape cultivars was performed according to the islands (Fig 3B) or sampling locations where the varieties *Verdelho* and *Arinto* occurred simultaneously (Fig 3C). This was not observed in the vineyard 12, where the percentage of *H*. *uvarum* isolates from *Arinto* grapes was of 83% while this species was not isolated from grapes of the *Verdelho* variety. However, in this vineyard, 100% of the isolates collected from *Verdelho* cultivar corresponded to *Hanseniaspora opuntiae*, representing a higher proportion of the *Hanseniaspora* genus on *Verdelho* cultivar in comparison to *Arinto*.

### Yeast species isolated from final stages of fermentations

From the 57 grape samples that were collected in five islands of the Archipelago, 40 spontaneous fermentations were achieved (Table 2). When the weight loss due to CO_2_ release during fermentation corresponded to 70 g L^-1^, a total of 1200 yeast isolates were collected at the final stages of fermentations (720 isolates from fermentations in 2009 and 480 from 2010). Thirty-one samples were predominantly fermented by *S*. *cerevisiae* (more than 75% of *S*. *cerevisiae* isolates in each sample). Ten fermentations were finished by non-*Saccharomyces* species (*Candida boidinii*, *C*. *glabrata*, *C*. *humilis*, *C*. *railenensis*, *Starmerella bacillaris*, *Hanseniaspora vinae*, *Issatchenkia hanoiensis*, *Kazachstania exigua*, *Kluyveromyces thermotoerans*, *Meyerozyma guilliermondii*, *Pichia kudriavzevii*, *P*. *terricola*, *Saccharomycodes ludwigii*, *Zygosaccharomyces bailii*). These species occurred together and in different proportions, with the exception of three fermentations that contained 100% of isolates of the species *C*. *boidinii*, *C*. *railenensis* or *C*. *glabrata*. The remaining 16 samples did not reach the final fermentative stage, since the weight loss was less than 65 g L^-1^ after 30 days of fermentation.

**Table 2:** Incidence of each yeast species (%) isolated from 40 spontaneous fermentations of grape samples collected on 5 islands of the Azores Archipelago (N*S*: Non-*Saccharomyces*; *Sc*: *S*. *cerevisiae;* BCT: Biscoitos; GRC: Graciosa Island; PLJ: Lajigos; PRL: remaining locations in Pico; SJG: S. Jorge Island; SMG: S. Miguel Island) from final stages of fermentations.

	Arinto da Terceira										Verdelho dos Açores										Terrantez do Pico		
	2009					2010					2009					2010					2009		2010	
	BCT	GRC	PLJ	PRL	SJG	BCT	GRC	PLJ	PRL	SJG	SMG	BCT	GRC	PLJ	PRL	SMG	BCT	GRC	PLJ	PRL	SMG	PRL	SMG	PRL
Nr. of fermentations	2	2	3	2	2	2	1	1	1	2	2	2	3	2	2	2	1	2	0	1	2	0	2	1
Nr. of yeast isolates	60	60	90	60	60	60	30	30	30	60	60	60	90	60	60	60	30	60		30	60		60	30
Nr. of species	5	2	7	1	2	1	1	1	1	1	2	3	1	3	2	5	1	1		1	1		1	1
*Candida boidinii*					50.0																			
*Candida glabrata*																	100							
*Candida humilis*																1.7								
*Candida railenensis*		50.0										45.0												
*Hanseniaspora vineae*	3.3		12.2											15.0										
*Issatchenkia hanoiensis*											1.7					40.0								
*Kazachstania exigua*																5.0								
*Kluyveromyces thermotolerans*												5.0												
*Meyerozyma guilliermondii*	33.3		5.6											50.0	1.7									
*Pichia kudriavzevii*			7.8																					
*Pichia terricola*			1.1																					
*Saccharomyces cerevisiae*	50.0	50.0	25.6	100	50.0	100	100	100	100	100	98.3	50.0	100		98.3	50.0		100		100	100		100	100
*Saccharomycodes ludwigii*	1.7																							
*Starmerella bacillaris*	11.7		25.6											35.0		3.3								
*Zygosaccharomyces bailii*			22.2																					

As shown in Table 2, higher species diversity was found in 2009 compared to 2010 (12 and 6, respectively). The species *S*. *cerevisiae* was predominant in both sampling years, corresponding to 64.5% and 87.5% of the total isolates obtained from the final stage of the fermentations performed in 2009 and 2010, respectively, independent of the grape cultivar and the island.

The species *C*. *railenensis*, *S*. *bacillaris*, *M*. *guilliermondii* and *H*. *vineae* occurred in two to five grape samples and each of these species was present only in two or three of the islands. The percentage of these species in each fermentation ranged between 6.7% and 100%. Less frequent species *I*. *hanoiensis* occurred in two fermentations from the same location (corresponding to 1.7% and 40% of the isolates). Some species occurred only in a single fermentation, representing between 66.7% and 100% of the isolates (*C*. *boidinii*, *C*. *glabrata* and *Z*. *bailii*) or between 10% and 24% of the isolates (*K*. *exigua* and *Lachancea thermotolerans*). Other species were rarely found, corresponding to one single isolate from a single fermentation (*C*. *humilis*, *P*. *terricola* and *S*. *ludwigii*).

From the 28 yeast species isolated in this study, listed in Tables 1 and 2, only five were found both in freshly crushed grapes and at the end of fermentations: *C*. *railenensis*, *S*. *bacillaris*, *I*. *hanoiensis*, *H*. *vinae* and *P*. *terricola*., For all but one of the grape samples, species occurring in the freshly crushed grapes did not occur at the end of the correspondent fermentation. One exception was observed for *P*. *terricola* that was isolated in both stages of the same fermentation.

### Comparison between the different sampling locations

**Fig 4:**
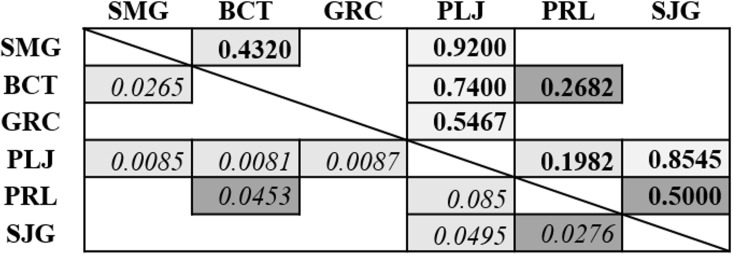
Analysis of similarity (ANOSIM) on square root transformed species incidences, using the software PAST [55]. Statistically significant differences in yeast community composition observed between islands/locations (SMG: São Miguel, BCT: Biscoitos, GRC: Graciosa, PLJ: Lajidos, PRL: remaining locations in Pico, SJG: São Jorge). *R*-values are indicated in bold, and *P*-values in italic. Light grey and dark grey boxes refer to comparisons made with yeast species obtained in 2009 and 2010, respectively.

Differences in community composition between sampling locations were tested by analysis of similarity (ANOSIM) on squared root transformed species incidences. Fig 4 shows R-values, indicative of the observed rank abundance difference between locations, ranging this value from -1 to +1, with positive and negative values indicating that communities differ or not, respectively.

Concerning the year of 2009, our results show that Biscoitos and Lajidos locations harbor communities that differ from the remaining locations of the archipelago. As shown in Fig 4, yeast communities collected in 2009 from the Lajidos location differed greatly from all islands of the Archipelago, with R-values ranging between 0.5467 (P = 0.0087) and 0.9200 (P = 0.0085). When comparing the Lajidos location with the “remaning locations in Pico”, despite a less significant difference, a positive R-value (0.1982; P = 0.085) indicates that they harbor different communities. In the first sampling year, significant differences were also found between yeast communities from Biscoitos and S. Miguel islands (R = 0.4320; P = 0.0265). However with lower significance, only positive R-values were observed from the comparison between Biscoitos with all other locations (except S. Jorge). In the second sampling year, the only significant differences between yeast communities were observed when comparing the “remaining location in Pico” with S. Jorge and Biscoitos (R = 0.5000 and 0.2682; P = 0.0276 and 0.0453), reflecting the predominance of *H*. *uvarum* in all grape samples, and the considerable decrease in species richness. Using this biodiversity index, together with Shannon index, similar results were obtained, being this analysis present in Supplementary data (S1 Fig, S1 Table).

## Discussion

A two-year sampling plan was designed and implemented in 13 locations, in five islands of the Azores archipelago. A total of 57 grape samples from three Azorean white grapevine cultivars were collected and 40 spontaneous fermentations were achieved. A total of 2910 yeast isolates were obtained and 28 yeast species were identified. We must caution that yeast isolates were obtained through selective conditions of growth that may differ from abiotic factors found in nature. Rarely occurring or slow-growing species may not have been detected, as the detection limit of our experimental approach is 3.3% (one species in 30 isolates). Also, when sequencing only one representative per grape sample (in a total of 450 profiles sequenced), some diversity could be lost. However, we consider that our approach allows a comparison of the yeast microbiota across vineyards and islands, even though it cannot provide a complete description of yeast community composition. Already in our previous work [56], we showed that this approach allowed the comparison between grape yeast communities. In adition, it was shown that the most representative genera belonging to these communities were comparable to the ones obtained in other continental areas. A fraction of the microbiota analyzed in this study was isolated after enrichment through must fermentation, allowing the comparison of the biodiversity of species enduring fermentation.

In agreement with most of the available bibliography, also in this study only non-*Saccharomyces* species were isolated from freshly crushed grapes and, for the first time, *C*. *carpophila* was encountered as an inhabitant of grape or wine-associated environments. To our knowledge so far, *Pichia cecembensis* has been isolated from grapes only in vineyards of the Azores Archipelago, from Pico and Terceira islands in one of our previous studies [57]. In the present study, this species was also found in Graciosa and S. Jorge. The wide occurrence of this species in the Azores (although in small proportions) may represent a particularity of the yeast biota from the vineyards of the archipelago. However, this species was only recently described [58], which might also explain the lack of previous findings in other grape ecosystems. The most representative species, *H*. *uvarum* and *M*. *pulcherrima*, are often reported as the predominant species on ripe grapes, in particular in regions with warmer climates [5,8,9,22,59–61]. *P*. *terricola* and *S*. *bacillaris* are also commonly reported as associated with grapes and wine environments [23,46,62–65]. However, in this study, both species occurred in higher proportions in comparison to other wine-producing areas. On the other hand, *H*. *opuntiae* has been less frequently reported as inhabitant of grape ecosystem [9,66–68], but in our study this species corresponded to 3.5% of the total isolates from freshly crushed grapes, despite being found only in 2 samples. Interestingly, in both samples *H*. *opuntiae* was predominant (more than 96% of the isolates) and *H*. *uvarum* was not found (while it was present in 82% of the remaining 55 grape samples). A similar correlation is reported by Settanni *et al*. [9], pointing to a possible antagonism between strains of both species. Globally, the remaining species (*C*. *inconspicua*, *C*. *quercitrusa*, *C*. *railenensis*, *H*. *vineae*, *I*. *hanoiensis*, *P*. *fermentans*, *P*. *membranifaciens*, *S*. *crataegensis*, *S*. *vini* and *W*. *anomalus*) were more rarely found on the archipelago, in accordance with most bibliographic references [5,65,69–72]. *Pichia terricola* was one of the most frequently found species in freshly crushed grapes, which is in agreement with recent reports that found this yeast in all stages of must fermentations [7,73]. In our study, only one isolate was obtained from final stages of the fermentations, suggesting that the isolated strains may have a low ethanol resistance or a weak ability to compete with other species. The remaining species isolated from finished fermentations were present in the correspondent freshly crushed grapes samples in proportions under the detection limit of our experimental approach (3.3%—one in 30 isolates). Only after enrichment through must fermentation could they be detected, with the frequencies and proportions generally found in similar studies on other geographic locations [4,5,7,46,63,74–76]. Among them, *S*. *cerevisiae* was predominant (in 75% of the fermentations), which is in agreement with the general observation that the isolation of *S*. *cerevisiae* from sound grapes, through direct plating methods has been rarely described (reviewed by [77]).

The effect of the non-*Saccharomyces* species on wine quality has been widely reported [71,78]. A possible linkage between yeast biota and a certain vintage, a specific grape cultivar and/or a particular wine-producing region may contribute to the typical sensory profile of the correspondent wines.

When considering the great differences found in the yeast biota between the two sampling years, we must stress that climatologically, the year of 2009 was characterized by the average conditions expected for the Archipelago, whereas unusually high frequency of precipitation was recorded in 2010. Rainfalls on the central and eastern islands were 20% and 60% higher, respectively, compared to the average values of the previous 30 years. In 2010, the adverse climatic conditions, led to a decrease of more than 80% of the average wine production. Globally, the yeast biota suffered a strong reduction in the species diversity from the first to the second sampling year (23 and 15 species respectively), which can be attributed to the pronounced climatic differences between the two sampling years. However, no data for the climatic variation between islands during grapes ripening period are available, which could impact those values obtained regarding yeast communities composition. In agreement with our results, some studies found a similar correlation between rainfall and yeast biodiversity [3,79], although other authors had reported a higher yeast biodiversity in rainy years [30,80]. This fact has been attributed to the effect of antifungal treatments that are carried out with greater intensity in years with higher precipitations [81]. In fact, some studies suggest that the usage of pesticides in vineyards decrease yeast biodiversity [82], however this is not universal [23,28,83,84]. Our previous studies [57] on yeast communities from abandoned vineyards suggested that the decrease of yeast diversity in rainy years is rather related to climatic conditions than to the usage of fungicides. Comparing both sampling years, a bigger decrease in species richness was observed in the yeast communities from the final stage of fermentations (from 12 to 6 species) than from the freshly crushed grapes (from 14 to 11 species).

Several studies suggest that particular yeast communities may be associated with certain grape cultivars [22,25,46,61,73,75,85,86], however no clear patterns or robust conclusions were achieved. Other authors did not find any correlation between grape cultivar and yeast communities composition [79,87]. We herein show that the yeast species *H*. *uvarum* tends to occur in higher proportions on grape samples from *Verdelho* than from *Arinto* cultivars. As described in the previous section, this was observed in both sampling years and in several locations and vineyards (Fig 3A–3C). Similar cultivar-specific yeast occurrences have been linked to varietal factors such as the thickness of the berry skin [88], that might be related to differences in berry composition, grape ripeness and sanitary condition at the sampling moment. However, this observation corresponds to a general tendency when the yeast biota was globally analyzed. The confirmation of a linkage between higher incidence of *H*. *uvarum* and the cultivar *Verdelho* needs further investigation. No differences were apparent between grape cultivars concerning the predominance of other species or concerning the presence of *S*. *cerevisiae*.

The geographical location and microclimatological conditions affects the composition of yeast communities isolated from grape and fermentations [22,46]. In agreement with this, our study revealed qualitative and quantitative variations in the yeast flora composition, between several locations from five islands of the Azores Archipelago. The species richness varied among geographical location, independently from the grape cultivar, sampling year, or fermentation stage. The highest yeast diversity was observed in the vineyards from S. Jorge, Biscoitos and Lajidos locations, having very particular environmental characteristics, corresponding to distinctive *terroirs*. The soils on these locations, covered by basaltic stone or solidified lava flows, have a strong effect on the microclimate at the grape level [89]. These environmental characteristics, together with an intensive viticultural activity, might explain the observed higher species richness. Differences were also observed concerning the occurrence of the predominant species on freshly crushed grapes from these locations. For example, *M*. *pulcherrima* showed a much higher incidence (*Arinto*: 95.6%; *Verdelho*: 83.3%) in the wine region Lajidos in comparison with the “remaining locations in Pico” (26%) in the sampling year 2009. The same was observed in the Biscoitos region of the Terceira Island concerning the proportion of *P*. *terricola*, which was considerably higher compared to the other islands of the archipelago. The statistical analysis of the yeast community composition revealed significant differences between those two locations and the rest of the archipelago. These differences were particularly accentuated in 2009, whereas the occurrence of atypical climatic conditions strongly affected grapevine growth and grapes development in the second sampling year. This may have affected yeast communities composition, and therefore differences between locations were less apparent.

This study is the first report on autochthonous yeast communities from the grapevine cultivars used in production of Azorean geographical indications wines. Considerable climate-associated variations were observed between vintages. Differences between cultivars were apparent and significant differences in yeast community composition were found between locations. In 2009 yeast communities found on freshly crushed grapes that were sampled from the locations of Lajidos and Biscoitos diverged greatly from the remaining locations of the archipelago.

Our observations strongly support the existence of a linkage between grape yeast communities and vineyards´ associated ecology. This fact is particularly true in vineyards with very distinctive environmental conditions.

## Supporting Information

S1 Fig PCA visualization obtained using average values of Shannon’s index and number of yeast species per sample or fermentation determined in six wine-producing areas of the Azores Archipelago (SMG–S. Miguel, BCT–Biscoitos, GRC–Graciosa, PLG–Lajidos, PRL–“Pico remaining locations”, SJG–S. Jorge). (DOCX)

S1 Table Shannon´s index and average number of species per sample calculated for each grape sample or fermentation, in 2009 and 2010 and six locations (SMG–S. Miguel, BCT–Biscoitos, GRC–Graciosa, PLG–Lajidos, PRL–“Pico remaining locations”, SJG–S. Jorge). (DOCX)
