# Supplementary material for: Association between Grape Yeast Communities and the Vineyard Ecosystems
Source: PLoS One. 2017 Jan 13;12(1):e0169883. doi: 10.1371/journal.pone.0169883 (PMC5234834; doi:10.1371/journal.pone.0169883)
Supplement: S1 Fig — (DOCX) [file pone.0169883.s001.docx]

Using data of Table S1 and for both parameters the average values were determined for each location on each sampling year and were used in a PCA analysis.


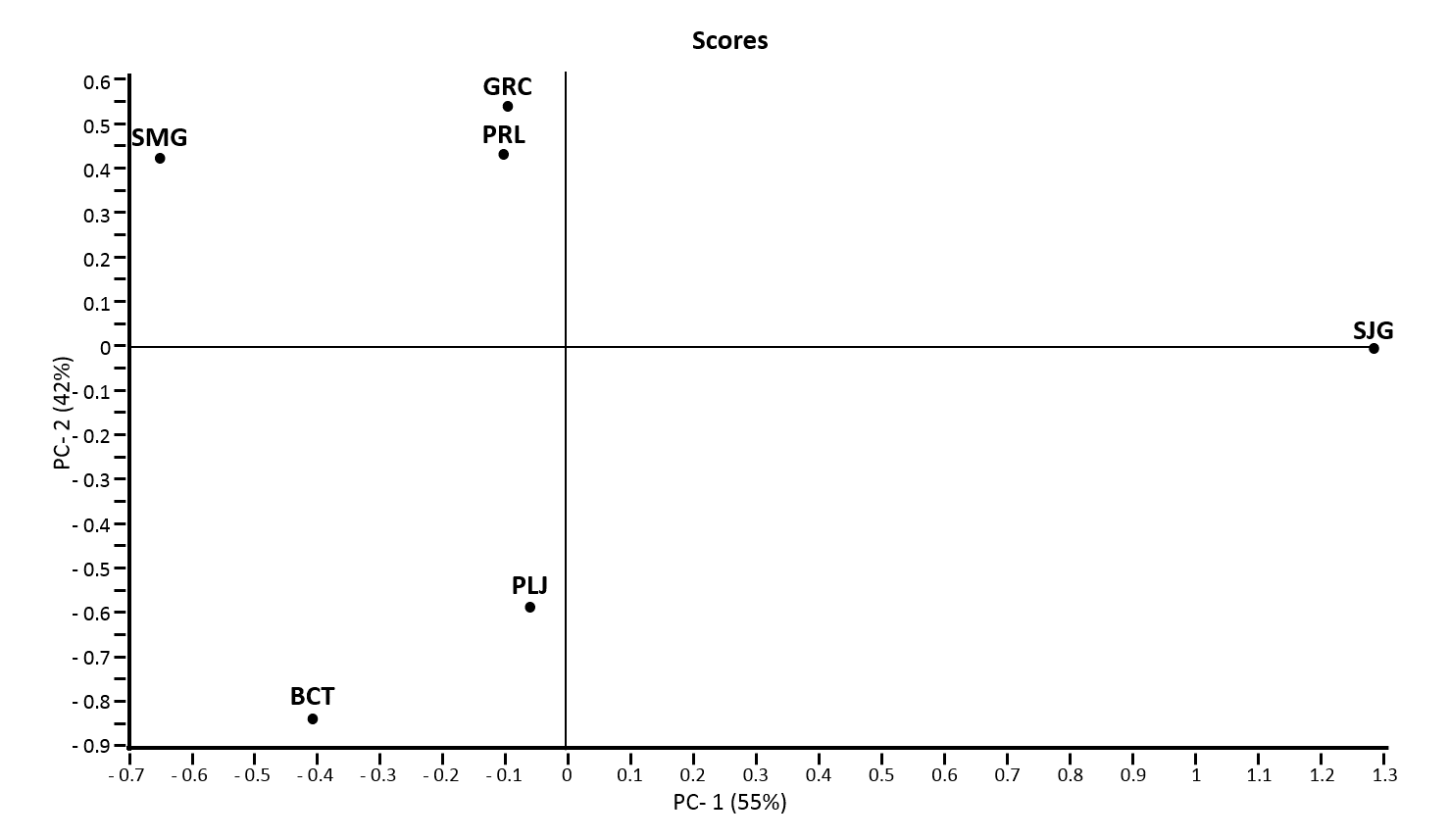


**A**


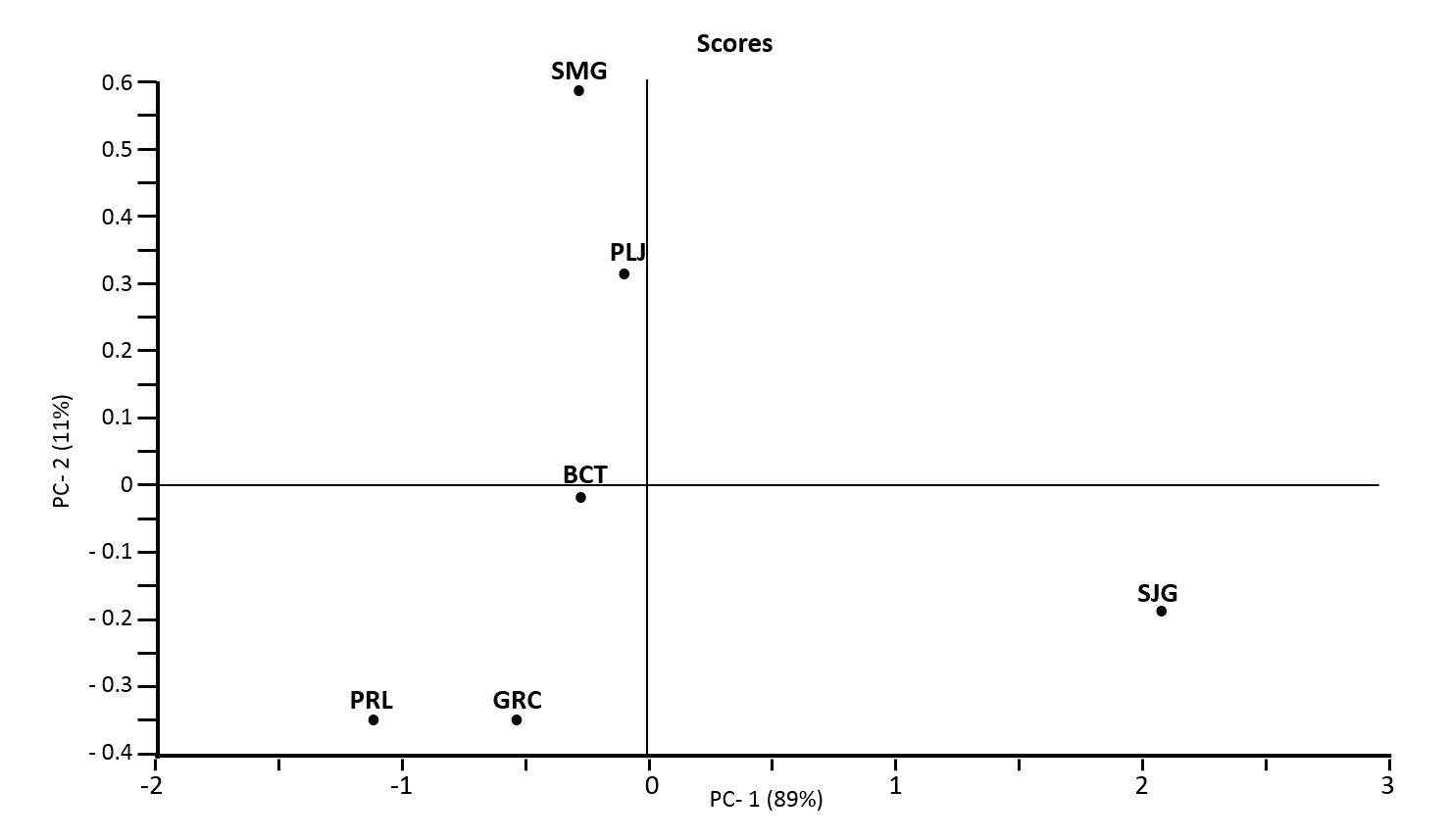


**B**

**Figure S1**: PCA visualization obtained using average values of Shannon’s index and number of yeast species per sample or fermentation determined in six wine-producing areas of the Azores Archipelago (SMG – S. Miguel, BCT – Biscoitos, GRC – Graciosa, PLG – Lajidos, PRL – “Pico remaining locations”, SJG – S. Jorge).
