# Supplementary material for: Association between Grape Yeast Communities and the Vineyard Ecosystems
Source: PLoS One. 2017 Jan 13;12(1):e0169883. doi: 10.1371/journal.pone.0169883 (PMC5234834; doi:10.1371/journal.pone.0169883)
Supplement: S1 Table — (DOCX) [file pone.0169883.s002.docx]

Shannon’s index, species richness and number of yeast species per sample or fermentation were calculated for the six wine-producing areas of the Azores Archipelago. The number of species per sample or fermentation was determined dividing the species richness of each location by the correspondent number of grape samples or fermentation. The Shannon’s index was calculated for each grape sample or fermentation.

**Table S1**: Shannon´s index and average number of species per sample calculated for each grape sample or fermentation, in 2009 and 2010 and six locations (SMG – S. Miguel, BCT – Biscoitos, GRC – Graciosa, PLG – Lajidos, PRL – “Pico remaining locations”, SJG – S. Jorge).

|  | Year | **2009** | | | | | |  | **2010** | | | | | |
| --- | --- | --- | --- | --- | --- | --- | --- | --- | --- | --- | --- | --- | --- | --- |
|  | Location | **SMG** | **BCT** | **GRC** | **PLJ** | **PRL** | **SJG** |  | **SMG** | **BCT** | **GRC** | **PLJ** | **PRL** | **SJG** |
| **Freshly crushed grapes** | Shannon’s index (average of all samples) | 0.31 | 0.43 | 0.33 | 0.31 | 0.43 | 0.59 |  | 0.26 | 0.50 | 0.21 | 0.43 | 0.10 | 0.93 |
|  | Average n° of species / sample | 0.80 | 0.80 | 1.17 | 1.20 | 1.14 | 2.50 |  | 1.25 | 1.20 | 1.00 | 1.40 | 0.43 | 3.50 |
| **Ended fermentations** | Shannon’s index (average of all samples) | 0.04 | 0.31 | 0.00 | 0.56 | 0.04 | 0.00 |  | 0.18 | 0.00 | 0.00 | 0.00 | 0.00 | 0.00 |
|  | Average n° of species / fermentation | 0.50 | 1.75 | 0.40 | 1.40 | 0.50 | 1.00 |  | 1.25 | 0.67 | 0.33 | 1.00 | 0.33 | 0.50 |
